# Co-design and Development of EndoSMS, a Supportive Text Message Intervention for Individuals Living With Endometriosis: Mixed Methods Study

**DOI:** 10.2196/40837

**Published:** 2022-12-09

**Authors:** Kerry Anne Sherman, Melissa Jade Pehlivan, Anna Singleton, Alexandra Hawkey, Julie Redfern, Mike Armour, Blake Dear, Tanya Jane Duckworth, Donna Ciccia, Michael Cooper, Kelly Ann Parry, Esther Gandhi, Sara A Imani

**Affiliations:** 1 Centre for Emotional Health School of Psychological Sciences Macquarie University Sydney Australia; 2 School of Health Sciences Faculty of Medicine and Health University of Sydney Sydney Australia; 3 Translational Health Research Institute (THRI) Western Sydney University Sydney Australia; 4 NICM Health Research Institute Western Sydney University Sydney Australia; 5 School of Psychological Sciences Macquarie University Sydney Australia; 6 School of Psychology Faculty of Medical and Health Sciences University of Adelaide Adelaide Australia; 7 Endometriosis Australia Sydney Australia; 8 Royal Prince Alfred Hospital Sydney Australia; 9 Australian College of Physical Education Sydney Australia

**Keywords:** text message, intervention, co-design, development, endometriosis, SMS, mHealth, self-management, mobile phone

## Abstract

**Background:**

Endometriosis, which affects 1 in 10 people assigned female at birth, is a chronic systemic inflammatory disease with a high symptom burden and adverse socioemotional impacts. There is a need for an accessible, cost-effective, and low-burden intervention to support individuals in managing their endometriosis condition.

**Objective:**

This study aimed to co-design and evaluate the acceptability, readability, and quality of a bank of supportive SMS text messages (EndoSMS) for individuals with endometriosis.

**Methods:**

In phase 1 of this mixed method design, 17 consumer representatives (individuals with endometriosis) participated across three 3-hour web-based (Zoom, Zoom Video Communications, Inc) focus groups. The transcripts were encoded and analyzed thematically. In phase 2, consumer representatives (n=14) and health care professionals (n=9) quantitatively rated the acceptability, readability, and appropriateness of the developed text messages in a web-based survey. All the participants initially completed a background survey assessing sociodemographic and medical factors.

**Results:**

Consumer representatives demonstrated diverse sociodemographic characteristics (Mage=33.29), varying in location (metropolitan vs rural or regional), employment, and relationship and educational statuses. Participants reached a consensus regarding the delivery of 4 SMS text messages per week, delivered randomly throughout the week and in one direction (ie, no reply), with customization for the time of day and use of personal names. Seven main areas of unmet need for which participants required assistance were identified, which subsequently became the topic areas for the developed SMS text messages: emotional health, social support, looking after and caring for your body, patient empowerment, interpersonal issues, general endometriosis information, and physical health. Through a web-based survey, 371 co-designed SMS text messages were highly rated by consumers and health care professionals as clear, useful, and appropriate for individuals with endometriosis. Readability indices (Flesch-Kincaid scale) indicated that the SMS text messages were accessible to individuals with a minimum of 7th grade high school education.

**Conclusions:**

On the basis of the needs and preferences of a diverse consumer representative group, we co-designed EndoSMS, a supportive SMS text message program for individuals with endometriosis. The initial evaluation of the SMS text messages by consumer representatives and health professionals suggested the high acceptability and suitability of the developed SMS text messages. Future studies should further evaluate the acceptability and effectiveness of EndoSMS in a broader population of individuals with endometriosis.

## Introduction

### Background

Globally, the inflammatory condition of endometriosis is estimated to affect 1 in 10 biological females [1]. Some countries report an even higher incidence, with estimates of prevalence in Australia being 1 in 9 over the reproductive lifetime [2]. Endometriosis is predominantly characterized by severe and chronic pelvic pain and painful periods (dysmenorrhea). Other common symptoms include painful sexual intercourse (dyspareunia), infertility, and abdominal bloating [3,4]. Migraine, fibromyalgia, irritable bowel syndrome, and chronic fatigue syndrome are frequently comorbid with endometriosis [5]. In the absence of a cure and with a chronic, high symptom burden, individuals living with endometriosis experience adverse effects on their socioemotional well-being and functioning [6-9] and poor overall well-being [10], characterized by diminished quality of life [11] and a high prevalence of psychological distress (eg, depression) [12,13]. Endometriosis-related impairments to physical and psychological functioning also impact the society through health care costs and lost productivity (approximately US $20,898 per individual per year [14,15]). Given the high symptom burden and adverse socioemotional consequences of endometriosis, there is a need for supportive interventions to help address the psychosocial impacts of living with this condition [16].

As a chronic condition, endometriosis demands ongoing self-management of physical symptoms (eg, pain and sleep hygiene) [17], which represents a substantial psychological and emotional burden on the individual [18,19]. The need for self-management may also serve as a constant reminder of one’s endometriosis diagnosis, exacerbating psychological distress [20]. From a psychological perspective, interventions designed to provide reassurance and support may assist in managing the self-regulatory aspects of living with endometriosis [21] and in diminishing psychological distress [22].

Previous research has highlighted that people with endometriosis have a tendency to be self-critical, particularly in relation to their body appearance and function [23], presenting a further barrier to maintaining good psychological health [24]. A growing body of literature indicates that self-compassion (the ability to view oneself in a kind, compassionate manner [25]) may act as a buffer in the face of adversity [26-29], reducing psychological distress and symptom burden among people with endometriosis [30]. This finding is consistent with other domains of women’s health (ie, breast cancer [31,32] and polycystic ovarian syndrome [33]). Perceived difficulties in forming and retaining social relationships [34-36] also contribute to high levels of psychological distress. Furthermore, endometriosis symptoms (eg, pelvic pain [3]) inhibit social functioning, particularly participation in social events [10,11,37].

Communicating with health professionals is another difficulty reported by many living with endometriosis [38,39]. This is in part because of a perceived normalization of endometriosis symptoms (eg, painful periods) by health professionals and associated feelings of menstrual stigma, which often leaves patients feeling unsupported in their endometriosis management [38,40,41]. For those actively seeking support, electronic, web-based resources are a preferred source of information [42]. However, despite there being websites from consumer-facing organizations [38] (eg, Endometriosis Australia) providing reliable information on a range of relevant topics [43], the feeling of being inadequately informed about endometriosis is commonly reported [41,42,44]. For many, not knowing where to find information or lacking the time to seek supportive information act as barriers to support [45]. Therefore, an intervention that helps direct those living with endometriosis to reliable web-based information on the nature and management of this condition is warranted.

In light of the many psychosocial challenges experienced by people with endometriosis, there have been worldwide calls for interventions to assist in managing difficulties with psychosocial functioning and to improve quality of life [16,46,47]. In particular, interventions providing tips and strategies to manage the psychological challenges of living with endometriosis that can be readily implemented by individuals living with endometriosis are highly valued. An approach ideally suited to achieve these aims are SMS text message interventions, entailing the delivery of brief SMS text messages through a mobile phone [48]. With the ubiquitous use of mobile phones worldwide [49], SMS text messages are a highly accessible, inexpensive, and convenient means of providing psychosocial interventions, as users can opt to receive messages at a preferred time [50]. SMS text messages have the added benefit of being “pandemic proof” in that they are not affected by government-imposed lockdowns or public health measures [22]. A key strength of SMS text messaging is its flexibility, allowing a range of content (within the 160-character limit), including psychoeducation, reminders (eg, to exercise or take medication), motivational messages, self-care (eg, stress management tips), and informative links to health-related internet sites [51]. The simple language format used in texts also increases their accessibility, irrespective of the reading level [52-55]. The ability to send SMS text messages anonymously may help minimize stigma and barriers to accessing health care [56], which are particularly evident in geographically isolated underserved populations [57]. With the increasing global uptake of smartphones [49], particularly among younger adults [58], supportive interventions using SMS text messaging platforms are likely to be ideally suited for the endometriosis population (ie, typical mean age 30, SD 7.50 years [11]); however, to date, no known SMS text message intervention has been developed for this population.

### Prior Work

Across a wide range of health contexts, SMS text message interventions have proven effective at facilitating health behavior change, such as smoking cessation [59-61], the adoption of exercise [61,62] and diet regimens [63], and in chronic disease prevention [61,63]. Critically, beyond a focus on health behavior change, SMS text message interventions are also being developed with the aim to provide psychological and emotional support [21,22,51,64]. For example, one psychologically supportive intervention designed to address the distress experienced by individuals living with the chronic condition diabetes uses SMS text messaging derived from positive psychology (eg, mindfulness and self-compassion) [65]. In this case, brief messages were designed to provide reassurance, encourage a positive outlook, and reflect on gratitude. Emerging evidence suggests that these psychologically focused supportive SMS text messaging interventions are very well tolerated and accepted [66], consistent with evidence from health behavior change–focused SMS text interventions [52,53].

### Study Aims

In sum, evidence across different health contexts suggests that SMS text messaging may be suitable to provide reassurance and support in managing the psychological and emotional self-regulatory aspects of living with endometriosis. Therefore, the aim of this study was to identify the needs and preferences of consumers living with endometriosis to inform the development of a suitable supportive SMS text messaging intervention. The involvement of consumers in this manner, known as co-design, is regarded as best practice for the development of health-focused interventions, as it ensures the relevance of the intervention and its content to the target audience [67-69]. For more than a decade, it has been best practice for researchers to collaborate with consumers in research [70]. Co-designed SMS text messages have been found to improve health-promoting behaviors (eg, smoking cessation and dietary changes [63,71]) in individuals with chronic conditions. Additionally, this study aimed to evaluate the initial acceptability, readability, and quality of the co-designed SMS text messages, in preparation for further evaluation and use in future studies. To achieve these aims, this mixed methods study entailed two phases: (1) the identification of consumer SMS text messaging needs and preferences through focus groups and (2) the development and review of the SMS text message bank.

## Methods

### Study Design

**Figure 1:**
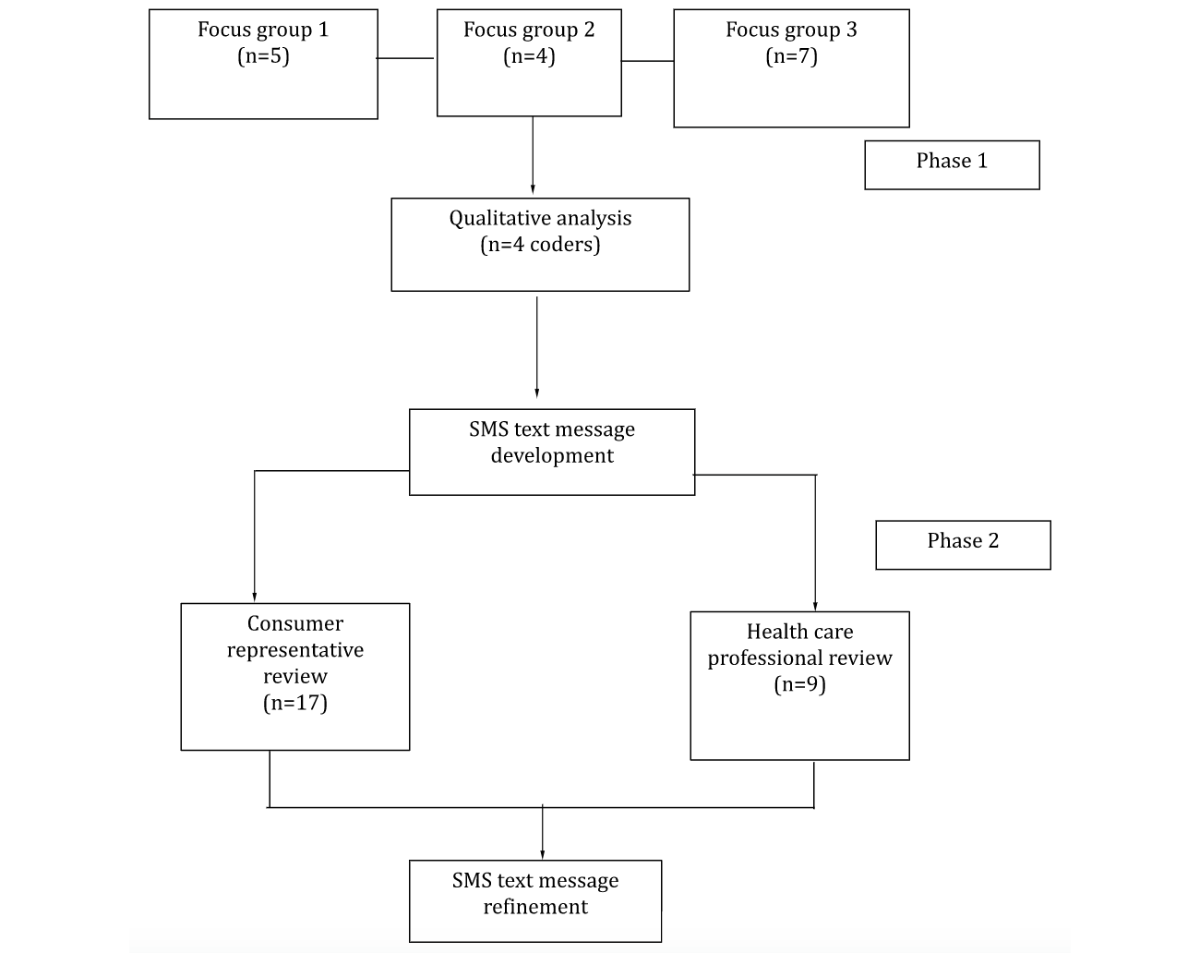
Study design diagram.

A 2-phase mixed methods approach was used with consumer representatives and researcher-clinician collaborators (Figure 1). Phase 1 involved a series of web-based focus groups with consumer representatives to ascertain the needs and preferences for a supportive SMS text messaging intervention. The focus groups were also used to co-design the development of a bank of SMS text messages to ensure the suitability of the end product [67-69]. In phase 2, the developed SMS text message bank was evaluated quantitatively and qualitatively by consumer representatives and health care professionals. Reporting for the focus groups and intervention development was based on the Consolidated Criteria for Reporting Qualitative Research (Multimedia Appendix 1) [72] and Template for Intervention Description and Replication (Multimedia Appendix 2) [73], respectively.

### Ethical Considerations

This study was approved by the Human Research Ethics Committee of Macquarie University (#52021963527729). All the participants provided web-based informed consent. The participants were informed that their confidentiality will be maintained, with only named investigators having access to their identifiable data. The participants were also informed that in the spirit of open science, their deidentified data would be made available in a public data store [74]. No incentives were provided for participation in the study.

### Phase 1: Consumer Focus Groups

Using co-design principles, the focus groups aimed to determine the structure and content of the SMS text messages.

#### Participants

A convenience sample of 1331 individuals living with endometriosis who had participated in prior endometriosis-related research of the lead investigator (KAS) were invited via email to participate. The eligibility criteria were as follows: (1) the participants should be aged ≥18 years, (2) diagnosed with endometriosis (self-reported), and (3) proficient in English. A citizen collaborator (TD) of the research team was also invited to participate in one of the focus groups. Of the 1331 individuals invited, 89 (6.7%) potential participants indicated their interest in the focus groups through return emails. Of these 89 individuals, 35 (39%) provided further details on their age and location (to facilitate the selection of a representative sample) and indicated their availability for the focus groups. Of the 35 participants who indicated availability for participation in the focus groups, 22 (63%) then completed a web-based informed consent form and a brief survey requesting further demographic (eg, age, education, employment, and location) and medical information (endometriosis treatments received and self-reported endometriosis severity, scored on a 5-point Likert scale from asymptomatic, 0, to severe, 4) via REDCap (Research Electronic Data Capture; Vanderbilt University). Consenting participants were then organized into 3 focus groups held over a week and spaced at least 1 day apart according to their preferred time and availability.

#### Facilitators

The focus groups were conducted by the lead investigator (KAS) and coauthor (AH), both of whom are experienced in qualitative research methods and research on women’s health. KAS is a female professor of health psychology with a PhD in psychology. AH is a female research fellow in women’s reproductive health with a PhD in critical public health. At the outset of the focus groups, KAS introduced herself as a professor with experience in women’s health research and lived experience with endometriosis, wanting to use her skills to give back to the endometriosis community. AH introduced herself as a researcher with interests in women’s health conditions, particularly menstrual disorders. In all the focus groups, there was only 1 lead active facilitator, with the second facilitator (AH) joining one of the focus groups conducted by the first facilitator (KAS) for quality assurance reasons. Also present in the focus groups, although not active in discussions, were a female research assistant (MJP) who took field notes during discussions and a female student research intern (EH) who was available to provide technological support as needed.

#### Consumer Focus Groups

A series of 3 web-based (via Zoom, Zoom Video Communications, Inc) 3-hour workshop focus groups were held in mid-July 2021 to accommodate most participant availabilities. Each focus group commenced with a brief presentation (approximately 15 minutes) outlining the background and aim of the SMS text message program to be developed and the objectives of the session. During the workshop, the participants engaged in discussions focused on the preferred structure (eg, number of SMS text messages per week and text message delivery time of the day and days of the week) and content themes of the SMS text messaging program. Before these discussions, the participants were prompted regarding the SMS text message structure (“How many times would you like to receive a text?” “Which days of the week would you like to receive a text?” “Would you like to have a text that comes at a particular time of the day?” and “Would you like to be able to respond to the text messages?”) and content themes. On the basis of prior endometriosis research [8,36,75-77] and SMS text messaging interventions developed by our research team [78,79], the participants were presented with five broad suggested topic areas along with example texts: (1) emotional health, (2) social support, (3) looking after and caring for your body, (4) general endometriosis information, and (5) physical health. The participants discussed the personal relevance of the proposed themes and were invited to suggest additional or alternate themes. The participants were also invited to draft their own example texts.

The 3 workshops were conducted in an iterative manner, building on the outcomes of the previous focus group sessions. At the conclusion of the third focus group, we reached data saturation, and, therefore, no more focus groups were needed. The focus groups were video recorded and transcribed using Zoom. The research intern (EG) reviewed the videos and corrected the transcripts for transcription errors. The participants were deidentified, were assigned an arbitrary number (eg, P1), and have been reported here accordingly to preserve their anonymity. Following the completion of the focus group transcription, four members of the research team (MJP, EG, SAI, and KAS) undertook a thematic analysis of these data [80] using a template approach [81] to identify the consumers’ broad themes of preferred SMS text messaging. The analysis was guided by the six-stage [82] inductive method entailing: (1) familiarization with the whole data set, where all the coders read these data once and then reread them while actively searching for patterns and meaning through note-keeping; (2) separate generation of initial codes through meaningful clustering of data; (3) comparison and refining of codes among coders to loosely identify potential themes—a process that was overseen by the senior member of the research team (KAS), ensuring consistency across codes and coders; (4) further discussion and refinement among the research team (MJP, EG, SAI, and KAS) for theme consolidation; (5) iterative naming of each theme, defining its singular focus, distinctness and extension of prior themes; and (6) integration of results through writing and selecting salient quotes.

### Phase 2: Text Message Development, Review, and Refinement

#### Text Message Development

Following the identification of the preferred themes of SMS text message content, the research team (including those with lived experience–KAS and TD) drafted SMS text messages across all themes. The guiding principles in developing these SMS text messages were to keep the text within the 160-character limit, attain a readability grade of no more than 8 on the Flesh-Kincaid scale, and have an approximately even distribution of messages across all themes. General endometriosis information and other medical information were based on best practice guidelines, as summarized on the Endometriosis Australia and Jean Haile’s Foundation websites, as well as clinical guidelines [83]. The SMS text messages were designed to provide support by drawing on the relevant psychological theory (Multimedia Appendix 3), such as the transactional model of stress [84] or self-compassion theory [25].

#### Participants

The research team, including clinicians (eg, gynecologists and psychologists) and academics, was invited to review the SMS text messages. All the consumer representatives and the citizen collaborator (TD) who participated in the focus groups, as well as the consumers who consented to the focus groups but were unable to attend them, were invited to review the SMS text messages via email.

#### Text Message Review

All the participants completed a web-based feedback survey via REDCap, with questions on participant demographics (eg, gender identity, profession, and years of experience) and approximately 40 draft SMS text messages (1-2 sentences each). Health care professionals reviewed the SMS text messages relevant to their area of expertise (eg, clinical psychologist researchers reviewed draft messages from the emotional health theme). Consumer representatives received a random selection of 30 draft SMS text messages, covering each of the different themes. For each draft text, the participants responded to 4 questions previously applied in SMS text messaging research [78], assessing the acceptability, readability, and appropriateness of the SMS text messages by rating them on a 5-point Likert scale (1=strongly disagree to 5=strongly agree; eg, “This message was easy to understand”). A free-text question for each item further gave the participants the opportunity to make suggestions for the improvement of the SMS text message. The scores on draft SMS text messages given by consumer representatives and health professionals were analyzed separately, with descriptive statistics (ie, means and range) generated for each question. The SMS text messages that received a mean rating of ≤2 on any of the 3 questions were reviewed by the research team and either revised or removed. Free-text suggestions for improvement were collated for each SMS text message. Through this process of editing and deleting, the message content was refined.

#### Readability

All the refined SMS text messages were evaluated for readability using the validated Flesch-Kincaid grade level [85], indicating the required education level to read the material, ranging from grade 1 (easiest) to grade 12 (most difficult).

## Results

### Phase 1: Consumer Focus Groups

#### Descriptive Statistics

In total, 22 individuals living with endometriosis (including the citizen collaborator—TD) completed the background demographic and medical survey. Subsequently, 23% (5/22) of participants were unable to attend the 3 focus groups (approximately 6-8 participants in each session) owing to a lack of availability, work commitments, inclement weather, or illness. The participants were aged between 21 and 48 years (mean age 33.29, SD 9.11 years), reported an average diagnostic delay of 10.85 years (SD 8.03 years), and had diverse sociodemographic characteristics (Table 1).

**Table 1:** Characteristics of consumer representatives.

Characteristics			Focus group and reviewer participants (n=17), n (%)		Reviewer only participants (n=3), n (%)
**Education**					
	High school education	4 (24)		1 (33)	
	Vocational or TAFE^a^	4 (24)		0 (0)	
	Undergraduate degree	5 (29)		1 (33)	
	Postgraduate degree	4 (24)		1 (33)	
**Employment**					
	Full time	11 (65)		3 (100)	
	Part time	2 (12)		0 (0)	
	Student	2 (12)		0 (0)	
	Unemployed	2 (12)		0 (0)	
**Location**					
	Metropolitan	11 (65)		2 (67)	
	Rural or regional	6 (35)		1 (33)	
**Relationship status**					
	Partnered	12 (71)		2 (67)	
	Not partnered	5 (29)		1 (33)	
**Treatment for endometriosis^b^**					
	Surgery	11 (65)		2 (67)	
	Hormonal medication	8 (47)		3 (100)	
	Pain medication	13 (76)		3 (100)	
	Other	6 (35)		2 (67)	
**Endometriosis severity**					
	Asymptomatic	0 (0)		0 (0)	
	Mild	3 (18)		0 (0)	
	Moderate	5 (29)		1 (33)	
	Severe	9 (53)		2 (67)	

^a^TAFE: Technical and further education.

^b^Categories not mutually exclusive.

#### Preferences for Content Themes

The 7 SMS text message themes and example quotes identified through template analysis are listed in Textbox 1. All 5 of the proposed SMS text message themes (ie, general endometriosis information, physical health, emotional health, social support, and looking after and caring for your body) were endorsed by the focus group participants as being relevant and helpful. In addition, two further SMS text message themes were identified: patient empowerment and interpersonal support. The consumers indicated that they require assistance to feel empowered in medical consultations and sought tips to help articulate their concerns so that health care professionals took them seriously. Tips and support regarding navigating intimate relationships, negotiating infertility and motherhood, and gaining support from loved ones and employers were also requested.

**Message content themes of focus groups with example quotes (focus groups consumers message content themes and quotes).**

**Emotional health**

- “My mental health has been greatly affected…so even just having reminders that it’s okay to seek help, psychologically and it doesn’t mean that there isn’t a physical issue and it doesn’t take away from endo[metriosis], but it’s important to treat both aspects, because they do have a lot in common” (P14)
- “Anyone who lives with the chronic pain knows that we do have mental health issues, to some degree or another. So the idea of a text message taking the stigma out of reaching out for help would be a perfect hint to do that” (P13)

**Social support**

- “Tips on how to utilize your support network to the best of your ability could be good…it’s important to touch base when you’re feeling bad but even celebrating all your little wins with people too” (P8)
- “A reminder to jump on Endometriosis Australia or something and just read someone else’s story or a reminder just to check in” (P14)

**Looking after and caring for your body**

- “People do tend to push themselves and I think it’s very important to remind people that you know your limits. And you know what your body can and can’t handle so to be careful about pushing yourself and putting yourself through that” (P1)
- “Setting some time to properly relax and be more self aware, like body and mind consciousness, can really help your body” (P2)

**Patient empowerment**

- “How to advocate for yourself when you’re speaking to doctors because a lot of doctors will just say, ‘No, that’s not right’ and move onto what they think without really listening to what your experiences are with the particular treatments that they’re suggesting, and you end up down a rabbit hole of doing the same things over and over again without getting any anywhere” (P17)
- “One thing that I found really helpful was, I just take notes on my phone. And whenever I’m having symptoms, write those down because I have a bit of anxiety around going to the doctor. And so I kind of forget what you know my symptoms are or forget half of it” (P10)

**Interpersonal issues**

- “I definitely have unsupportive family members and it’s like, really hard to get them to, to talk to them about this [endometriosis]” (P2)
- “I think education or tips on how to manage and communicate with your employer about endo[metriosis]” (P16)
- “Painful sex...just kind of some tips around that, as well, because yeah it’s yeah, it’s a big thing and that can lead to like low self-esteem, confidence and things like that” (P2)
- “There’s not a lot of information about the impact of endometriosis on romantic relationships, and that it’s really important people are given information on how to navigate having endometriosis in a romantic relationship because it does have a strain particularly if it’s impacting sex” (P17)

**General endometriosis information**

- “I think it would be helpful just to kind of give some guidelines as to what’s actually true about the disease so that you know you’re kind of more equipped because when this all started, I was only 13” (P12)
- “I liked the idea of [general endometriosis information] being for beginners. But also I’ve had endometriosis for about 29 years and I’m still learning things about it” (P14)

**Physical health**

- “Definitely for me, I think, pain, like the physical side of it. Having tips on pain management. I think would be really useful” (P11)
- “Recipe tips because I know when I’m absolutely wrecked, the last thing I want to do is think about what to cook so if there’s a recipe suggestion that comes through...something that’s easy and quick because if you’re struggling, the last thing you want to do is cook for an hour” (P16)

#### Preferences for the Structure of the Text Message Program

The consumers could see the value in a SMS text messaging program that provides them with information on endometriosis and tips to manage their daily lives in a highly convenient and accessible way:

I’ve been searching for things to try to help me more in regard to learning more about endo[metriosis] and to actually have that information given to me, as opposed to me having to actually find it would be really good.

P5

In particular, the consumers wanted to be linked up with web-based resources that they could access from their phone:

The whole point of this is that you are on your phone, so trying to give you links to other resources that are also on your phone could really help.

P16

The consumers unanimously agreed that the most appropriate frequency would be 4 SMS text messages per week, covering a variety of topics:

I think the thing is if you are getting messages with like different topics or different things it’s not necessarily going to feel like four a week.

P16

A range of preferences regarding the timing of the SMS text message delivery was evident: randomly during the week or the weekend (“I’m happy for it [the day of the week] to be random. It doesn’t really bother me.” [P17]); a midweek text (“My first thought is that hump day Wednesday is a great day to get a pickup message from someone.” [P13]); and a weekend text to assist with recovery after exerting themselves, for example, going out with friends and drinking (“I feel like I get more pain during the weekend sometimes depending what I do.” [P2]).

Views on at what time of the day to receive texts were also mixed, with some consumers preferring to receive the texts at night (“Everyone’s got stuff to do to during the day. I think it’s the nights that kind of get hard as well.” [P11]) and others preferring a morning text to help prepare them for the day (“I’m definitely a night person myself, but I need a message in the morning because it’s just hard to get out of bed when I feel rubbish.” [P4]).

The consumers had differing views about whether they would prefer 1- or 2-way communication (ie, option to text back), with some indicating that 2-way communication would add an element of pressure:

I think I prefer not responding. Honestly, just so that I don’t have that pressure.

P11

Two-way communications from chatbots were also strongly disliked by the consumers:

I guess two way, but I want to know who is on the other side, if a robot is going to send a message back to me then I don’t want to speak to a robot.

P2

Following the discussions, all the focus group participants concluded that one-way communications may be the most practical. Regarding the formality of the texts, the consumers indicated that they would prefer causal greetings (eg, “hey”) using their names with inclusive terminology (ie, avoid gender-specific pronouns).

A total of 376 messages were co-designed by the research team after the feedback from the focus group workshops, across seven topic areas, namely the topic areas initially suggested by the researchers—emotional health (n=52, 13.8%), looking after and caring for your body (n=52, 13.8%), social support (n=30, 8%), general endometriosis information (n=76, 20.2%), physical health (n=79, 21%)—and those suggested by the consumer representatives—patient empowerment (n=37, 9.8%) and interpersonal issues (n=50, 13.3%). On the basis of the feedback provided in the focus group workshops, the SMS text messages were designed to be delivered at a rate of 4 messages per week (on a mix of weekdays and weekends) and semicustomized to be delivered at their preferred time of the day (eg, morning, daytime, or nighttime), using their name.

### Phase 2: Text Message Review and Refinement

A total of 14 participants from the consumer focus groups and an additional 3 consumer representatives completed the SMS text message review (Table 1). In addition, 9 health care professionals with an average of 10.11 (SD 6.86) years of experience completed the SMS text message review. Most of them identified as a female (6/9, 67%) and a health care researcher (6/9, 67%), with 33% (3/9) identifying as a researcher clinician.

The 376 co-designed messages were reviewed at least twice: once by a consumer representative and once by a health care professional. Overall, the consumers and health care professionals either agreed or strongly agreed that the SMS text messages were clear, useful, and appropriate for individuals with endometriosis (Table 2). In total, 7.7% (29/376) of messages received a score of 1 or 2 for at least one of the acceptability questions, warranting further review and revision by the research team.

For 41.8% (157/376) of the SMS text messages, the reviewers left free-text response comments suggesting improvements. Free-text feedback themes from health care professionals (120 in total) focused predominantly on website links and the grammar and clarity of the messages. Consumers’ feedback (51 in total) focused on bolstering messages with resources (eg, links and tips) and ensuring that there was no insinuation of blame. On the basis of this feedback, of 376 messages, 6 (1.6%) messages were removed, 116 (30.9%) were revised to reflect reviewer feedback, and 1 (0.3%) new message was created, resulting in 371 co-designed SMS text messages, with an average Flesch-Kincaid readability score of 7 (providing high accessibility [86]). Table 3 displays the distribution of SMS text messages per theme, their Flesch-Kincaid grade, and example text messages for each.

**Table 2:** Consumer and health professional ratings of text messages.

Statements	Consumer representative ratings, mean (SD; range)	Health care professional ratings, mean (SD; range)
This message was easy to understand	4.40 (0.76; 2-5)	4.73 (0.46; 4-5)
The information provided in this message is useful	4.31 (0.82; 2-5)	4.57 (0.69; 1-5)
This message is appropriate for individuals with endometriosis	4.40 (0.78; 2-5)	4.63 (0.63; 1-5)

**Table 3:** Breakdown of readability and examples of SMS text messages for each theme.

Theme and example		Domain	Messages, n (%)		Flesch-Kincaid grade, mean (SD)	
**Emotional health**				52 (14)		5.51 (2.58)
	Hey <name>, it’s okay to reach out and talk to someone about how you're feeling. Having endometriosis is tough and can impact every part of your life.	Social support enhancing				
	Worried all the time? Why not try setting aside 10 minutes a day to write down whatever is troubling you, rather than letting it interrupt your day.	Coping strategy				
**Social support**				30 (8.1)		5.44 (2.77)
	Hey <name>, remember to celebrate your daily wins with the people who support you!	Social support enhancing				
	Don’t feel like getting out and about lately? Why not try connecting with friends or family from the comfort of your own home. You could FaceTime, call, or chat online.	Coping strategy				
**Looking after and caring for your body**				49 (13.1)		6.31 (2.85)
	Hey <name>, when was the last time you felt really relaxed? Try to do something you find relaxing today, whether it’s reading a book or watching the sunset!	Coping strategy				
	Hey <name>, everyone has their own limits. Try not to compare yourself with others. You are on your own journey!	Coping strategy				
**Patient empowerment**				37 (10)		6.55 (2.63)
	Seeing your health care professional soon? It might help to keep a journal of your symptoms and bring this along with you to your appointment.	Coping strategy				
	There’s nothing wrong with getting a second or even third opinion on your condition. They can help you and your GP^a^ better understand what’s going on for you!	Education				
**Interpersonal issues**				51 (13.8)		7.06 (2.82)
	Pain during sex can change throughout your cycle. Try and find a time that works best for you!	Education and oping strategy				
	Hey <name>, having a bad flare up? Don’t be afraid to ask your employer for some flexibility to work from home or some time off work.	Coping strategy				
**General endometriosis information**				75 (20.2)		7.97 (2.16)
	Hi <name>, if you’re experiencing heavy bleeding, you may also be feeling more tired than usual. Remember that it’s okay to take time and rest.	Education				
	Hi <name>, heard something about endometriosis that you aren’t sure about? Double check the facts here <link to Endometriosis Australia fact buster information sheet>	Education				
**Physical health**				77 (20.8)		6.15 (1.95)
	Hi <name>, if you’re feeling okay today, why not try and get some exercise in? Just parking further away from the shops or work can get your steps up!	Symptom management				
	Hi <name>, if you’re in pain, you might find it hard to eat. Berries can be a great choice if you’re wanting a snack, due to their anti-inflammatory effects.	Symptom management				

^a^GP: general practitioner.

## Discussion

### Principal Findings

This study aimed to identify the needs and preferences of consumers living with endometriosis for an SMS text message–based supportive intervention. Consistent with prior research indicating a need for supportive interventions for endometriosis populations [16,20], our findings identified a strong desire for an SMS text message intervention that would provide both access to reliable information about endometriosis and its management and support. Consumers desired tips and strategies regarding physical and emotional health, social support and interpersonal issues, looking after and caring for their body, patient empowerment, and general endometriosis information. In particular, SMS text messaging was seen positively, with consumers expressing a desire to have supportive messages sent to their phones for easy access. It is likely that for individuals living with endometriosis who frequently experience fatigue and other high symptom burdens [3,5], having this supportive message readily available through the convenience of a mobile phone is particularly appealing. This points to the acceptability of mobile health interventions for individuals with endometriosis.

The need for psychological and emotional support was strongly evident in the focus groups, with 71% (5/7) of the themes reflecting this. Although various reviews have documented the psychological and socioemotional tolls associated with endometriosis [6,12,34,87,88], little research has been dedicated to developing supportive interventions that address these concerns [89,90]. To date, most of the psychological or supportive interventions developed for individuals with endometriosis are of low quality, lacking evidence-based protocols for replicability and being evaluated under nonrigorous conditions (eg, in the presence of confounding variables) [16,90]. In particular, our qualitative analysis revealed several distinct aspects of psychosocial functioning with which individuals with endometriosis required assistance. In line with prior research, individuals with endometriosis may have difficulty with self-compassion [23], and the consumers sought reminders to be kind to themselves, whether this be through physical acts (eg, self-care activities) or positive self-talk.

Similarly, consistent with extant literature [34-36], individuals with endometriosis expressed an interest in receiving support in maintaining social relationships through prompts to reach out or seek alternative methods of connection (eg, on the web) when facing debilitating symptoms. A need for support in managing interpersonal issues that went beyond ongoing relationship maintenance and general social support was also reported. In line with previous research [38,39], the participants reported a range of interpersonal conflicts as a result of endometriosis in their professional (eg, with employers and teachers) and more intimate relationships (eg, with family members and romantic partners), which they required assistance with. Furthermore, individuals with endometriosis expressed a desire to have more meaningful communications with health care professionals and sought support in advocating for their health care needs. This reflects prior research that documents the difficulties individuals with endometriosis face in their relationships with health care professionals [38,39].

The consumers preferred that the SMS text messages be delivered in a variety of ways (eg, at different times during the day) to suit their diverse lives. This highlights a need for creative, adaptive, and tailorable intervention strategies. Another aspect that customization was required for was medication adherence. Some consumers expressed an interest in receiving SMS text messages designed to assist them in adhering to their medication for endometriosis. EndoSMS offers a novel solution, enabling tailoring to deliver messages at consumers’ convenience with customization of the message content (eg, inclusion and exclusion of medication adherence texts).

Promisingly, the co-designed SMS text messages were rated highly overall in acceptability and suitability for an endometriosis population in the SMS text message review by both consumer representatives and health care professionals. Furthermore, the messages were considered easy to understand, with anyone with education until at least seventh grade able to read the messages. These preliminary results suggest that EndoSMS may be highly suitable for an endometriosis population; however, further evaluation in terms of the intervention’s acceptability and effectiveness in a broader population of individuals with endometriosis is needed.

### Strengths and Limitations

This mixed methods study co-designed and evaluated the acceptability of a supportive SMS text message intervention for individuals with endometriosis. To our knowledge, this is the first mobile health intervention to be designed for individuals with endometriosis. The strengths of this study include the involvement of consumer representatives in the co-design and evaluation of the intervention, ensuring tailoring to meet the needs of its end users in an appropriate and sensitive manner [67-69]. Furthermore, the inclusion of health care clinicians and researchers in the evaluation of the SMS text messages ensured the content validity and suitability for an endometriosis population. Consumer representatives demonstrated diverse sociodemographic characteristics, bolstering the generalizability of the EndoSMS program across different users. However, it should be noted that EndoSMS is designed for an Australian audience, and piloting of the program’s feasibility is required to ensure its suitability for international use. In addition, although there was a high level of acceptability for the SMS text messages evident from the consumer representative ratings, it should be noted that many of these participants also informed the development of these messages, which perhaps explains the favorable ratings. Further research on the acceptability and helpfulness of the SMS text messages and EndoSMS program is needed to determine the suitability of the intervention for a broader endometriosis population. In particular, a pilot randomized controlled trial is needed to confirm that the SMS text messages are indeed supportive.

### Conclusions

This study entailed the co-design of EndoSMS, a supportive SMS text message program for individuals with endometriosis, and investigation regarding its preliminary acceptability. By ascertaining the needs and preferences of a diverse group of consumer representatives, EndoSMS was co-designed to better support individuals with endometriosis. Furthermore, the initial acceptability evaluation by consumers and health care professionals was highly favorable. Further evaluation of EndoSMS is required to confirm its acceptability and effectiveness in a broader endometriosis population.

## Appendix

Multimedia Appendix 1 Consolidated Criteria for Reporting Qualitative Research checklist.

Multimedia Appendix 2 Template for Intervention Description and Replication checklist.

Multimedia Appendix 3 Example text messages across identified themes.

## Abbreviations

REDCap: Research Electronic Data Capture

## Data Availability

The identified group-level data sets generated during and analyzed in this study are available from the Open Science Framework (https://osf.io/m7bu8).
