# Supplementary material for: Co-design and Development of EndoSMS, a Supportive Text Message Intervention for Individuals Living With Endometriosis: Mixed Methods Study
Source: JMIR Form Res. 2022 Dec 9;6(12):e40837. doi: 10.2196/40837 (PMC9789499; doi:10.2196/40837)
Supplement: Multimedia Appendix 3 [file formative_v6i12e40837_app3.docx]

Multimedia Appendix File 3 – Example text messages across identified themes

| Theme | Example | Domain | Theory |
| --- | --- | --- | --- |
| General endometriosis information | Hi <name> Are you experiencing pelvic or abdominal pain? Try using some heat – a heat pack, hot water bottle, or even a cloth soaked in warm water can help! | Symptom management | HAPPA^a^, CSM^b^, |
|  | It may be difficult to find an endometriosis specialist near you. To make it easier, check out Endometriosis Australia: <link to Endometriosis Australia link page> | Education | TPB^c^ |
|  | Hey <name> Are you interested in volunteering with Endometriosis Australia? Check out ways to get involved here: <link to Endometriosis Australia how you can help page> | Social support enhancing | HAPPA, TPB |
|  | Hi <name> Symptoms of endometriosis can be different for different people. Check out some common symptoms here: <link to Jean Hailes symptoms and causes page> | Education | TSM^d^ |
|  | Hi <name> Have you heard different things about what symptoms you may experience during pregnancy? Not everyone is the same, consider talking to your ob-gyn | Education | CSM, HAPPA |
| Physical health | If you're in a lot of pain, exercise may be the last thing on your mind. Listen to your body and remember, even getting up for a heat pack is movement. | Symptom management | HAPPA, TSM |
|  | Hi <name>, including vegetables in your diet can sometimes be hard, especially if there’s a lot of prep work. Try frozen pre-cut vegies as a time-saving option! | Education | HAPPA, TPB |
|  | Hi <name>, have you noticed any food that may reduce a flare? Next time you eat something that reduces the pain perhaps make a note on your phone as a reminder. | Symptom management | TSM |
|  | Sleeping well? While alcohol may help you fall asleep initially, it can disrupt the sleep cycle. For a better sleep, try limiting alcohol late at night. | Education | HAPPA, TPB |
| Emotional health | Did you know most schools/universities have on-site counsellors and many workplaces provide EAP programs - all free and confidential counselling services | Education | HPM^e^, TSM |
|  | Mental health support is more than 'seeing someone’! There are plenty of evidence-based online free resources. Check out This Way Up <link to This Way Up home page> | Coping strategy | TSM |
|  | Hey <name> , it's ok to not feel 100% all of the time. We all have our good and bad days. | Coping strategy | TSM, SC |
|  | Have you lost interest in activities which used to be enjoyable for you? This could be a warning sign of depression. Speak to your GP for more information | Education | HAPPA, CSM |
| Social support | Have you considered joining an endo support group to meet and talk to people with similar experiences? Click here to find one: <link to Endometriosis Australia support groups page> | Social support enhancing | TSM |
|  | Hey <name>, having support from family and friends can make all the difference. If you need support, don’t be afraid to ask. | Social support enhancing | TSM |
|  | Hey <name>, don't be afraid to ask your support system if you need help with day-to-day activities. These can be things like chores or shopping! | Symptom management | TSM |
|  | Hey <name>, had to cancel some plans today because of pain? Don't sweat it. You've done well to put your health first! | Symptom management | SC^d^, TSM |
| Looking after and caring for your body | Setting aside 15 minutes of your day to do something you love can help lift your mood - what do you love doing, <name>? | Coping strategy | SC, TSM |
|  | <name> are you finding it difficult to complete your tasks today? Consider breaking them down into smaller parts as this can make it more manageable. | Coping strategy | TSM |
|  | Self-care is more than just spa days and massages. Self-care can be as simple as staying hydrated and eating well. Can you show yourself some love today? | Coping strategy | SC |
|  | Remember to be kind to yourself! Think of three things you're thankful that your body can do | Coping strategy | SC |
| Patient empowerment | Ever felt unsure about what your GP/ specialist is recommending for you? It's okay to speak up and ask more questions and to let them know of any concerns. | Education | HAPPA |
|  | Hey <name>, don’t be afraid to let your GP/specialist know what's been going on for you lately. Your health care professional is there to help you. | Symptom management | HAPPA, TSM |
|  | Want to track your endometriosis symptoms but are not sure what to track? Check out this free pain/symptom tracker worksheet <link to Endometriosis Australia pain tracker worksheet> | Symptom management | HAPPA, TPB |
|  | Doing some of your own research on endometriosis using trusted sources can be empowering! Check out Endo AU as a trusted source <link to Endometriosis Australia what is endometriosis page> | Education | HAPPA, TPB |
|  | Hi <name>, bringing a trusted person to your health care appointments can help you feel supported and confident. | Coping strategy | TSM |
| Interpersonal issues | Sex can be a difficult subject to discuss. However, good communication with your partner about your thoughts and feelings is a really important start. | Social support enhancing | TSM |
|  | Intimacy is more than just physical. Building emotional intimacy with your partner is just as important. Why not take a trip down memory lane together. | Coping strategy | TSM |
|  | Hey <name>, endometriosis does NOT always cause infertility. Talk to your healthcare professional about your unique circumstances. | Education | HPM, CSM |
|  | Hey <name>, are you struggling with a new season of baby announcements? You don't have to hold it in, talk to a trusted friend. | Coping strategy | TSM |

^a^Health Action Process Approach

^b^Common-Sense Model of Self-Regulation

^c^Theory of Planned Behaviour

^d^Transactional Model of Stress

^e^Health Promotion Model

^f^Self-Compassion
